# Cerebral blood flow and neurocognition in patients undergoing transcatheter aortic valve replacement for severe aortic stenosis

**DOI:** 10.1093/ehjopen/oead124

**Published:** 2023-12-07

**Authors:** Ronald M Lazar, Terina Myers, Toby I Gropen, Massoud A Leesar, James Davies, Adam Gerstenecker, Amani Norling, Marykay A Pavol, Randolph S Marshall, Susheel Kodali

**Affiliations:** Department of Neurology, University of Alabama at Birmingham, 1720 7th Avenue South, SC650K, Birmingham, AL 35294, USA; Department of Neurology, Columbia University Irving Medical Center, 710 W168th Street, NewYork, NY 10032, USA; Department of Neurology, University of Alabama at Birmingham, 1720 7th Avenue South, SC650K, Birmingham, AL 35294, USA; Department of Neurology, University of Alabama at Birmingham, 1720 7th Avenue South, SC650K, Birmingham, AL 35294, USA; Department of Medicine, University of Alabama at Birmingham, Birmingham, AL, USA; Department of Surgery, University of Alabama at Birmingham, Birmingham, AL, USA; Department of Neurology, University of Alabama at Birmingham, 1720 7th Avenue South, SC650K, Birmingham, AL 35294, USA; Department of Neurology, University of Alabama at Birmingham, 1720 7th Avenue South, SC650K, Birmingham, AL 35294, USA; Department of Neurology, Columbia University Irving Medical Center, 710 W168th Street, NewYork, NY 10032, USA; Department of Neurology, Columbia University Irving Medical Center, 710 W168th Street, NewYork, NY 10032, USA; Department of Medicine, Columbia University Irving Medical Center, NewYork, NY, USA

**Keywords:** Aortic valve stenosis, TAVR, Cerebral blood flow, Neurocognition

## Abstract

**Aims:**

Aortic valve stenosis (AS) results in higher systolic pressure to overcome resistance from the stenotic valve, leading to heart failure and decline in cardiac output. There has been no assessment of cerebral blood flow (CBF) association with neurocognition in AS or the effects of valve replacement. The goal was to determine if AS is associated with altered cerebral haemodynamics and impaired neurocognition, and whether transcatheter aortic valve replacement (TAVR) improves haemodynamics and cognition.

**Methods and results:**

In 42 patients with planned TAVR, transcranial Doppler (TCD) assessed bilateral middle cerebral artery (MCA) mean flow velocities (MFVs); abnormality was <34.45 cm/s. The neurocognitive battery assessed memory, language, attention, visual–spatial skills, and executive function, yielding a composite *Z*-score. Impairment was <1.5 SDs below the normative mean. The mean age was 78 years, 59% Male, and the mean valve gradient was 46.87 mm/Hg. Mean follow-up was 36 days post-TAVR (range 27–55). Pre-TAVR, the mean MFV was 42.36 cm/s (SD = 10.17), and the mean cognitive *Z*-score was −0.22 SDs (range −1.99 to 1.08) below the normative mean. Among the 34 patients who returned after TAVR, the MFV was 41.59 cm/s (SD = 10.42), not different from baseline (*P* = 0.66, 2.28–3.67). Post-TAVR, average Z-scores were 0.17 SDs above the normative mean, not meeting the pre-specified threshold for a clinically significant 0.5 SD change.

**Conclusion:**

Among patients with severe AS, there was little impairment of MFV on TCD and no correlation with cognition. Transcatheter aortic valve replacement did not affect MFV or cognition. Assumptions about diminished CBF and improvement after TAVR were not supported.

## Graphical Abstract

**Graphical Abstract:**
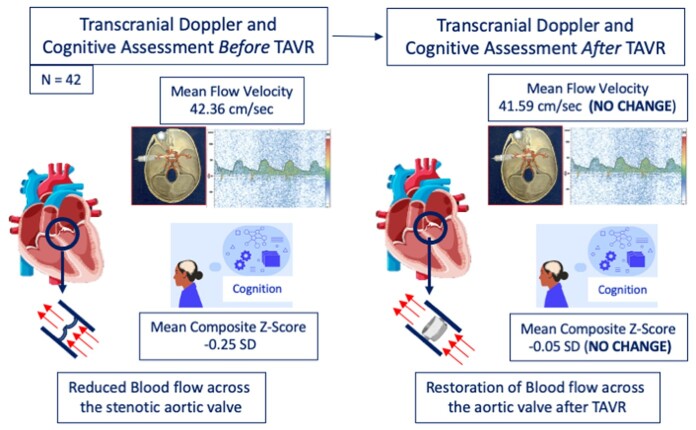
The relationship of cognition (*Z*-scores) to cerebral haemodynamics (mean flow velocity). Abnormality: ≥1.50 SD below normative means.

## Introduction

Transcatheter aortic valve replacement (TAVR) is a treatment for severe aortic stenosis (AS).^1^ Previous studies of TAVR and the brain have involved ischaemic injury^2^ but lacked cerebral haemodynamics [cerebral blood flow (CBF)] and its relationship with cognition. We wanted to determine if TAVR benefits the brain.

## Methods

### Patients

Patients enrolled at the University of Alabama at Birmingham Medical Center and the Columbia University Irving Medical Center. Institutional review boards approved this study; participants signed informed consent.

Inclusions included severe AS (mean pressure gradient >40 mmHg, peak velocity >4.0 m/s, and valve area <1.0 cm, either at rest or with dobutamine); planned TAVR; and English fluency. Exclusions were prior major stroke, known carotid disease, uncontrolled vascular risk factors, severe pulmonary disease, and inadequate sonographic windows for transcranial Doppler (TCD).

Participants underwent the Mini-Mental State Exam for frank dementia; ineligibility was ≤23/30.

### Measurement of haemodynamics

Patients underwent transthoracic echocardiography and had severe AS per the American College of Cardiology valvular heart disease guidelines.^3^ The TCD equipment was certified by the Intersocietal Accreditation Commission. Using a standard headframe, middle cerebral arteries were located through the temporal windows bilaterally at a depth of 50–56 mm using 2 MHz probes. After stability of waveforms, mean flow velocity (MFV) was acquired bilaterally over the next 2 min and averaged. If bone windows could not be obtained for either middle cerebral artery (MCA), values came from the remaining MCA. Abnormal MVFs were ≤34.5 cm/s.^4^

### Neurocognitive testing

There were nine standardized neuropsychological tests used previously in the Sentinel trial for TAVR^5^ and aligned with recent Neurologic Academic Research Consortium guidelines.^6^ The test battery (*Table 1*) was scored centrally, blinded of TCD findings.

**Table 1:** The neuropsychological test battery

Neurocognitive test	Domain
Trail Making Part A	Attention
Digit Span	Attention
Trail Making Part B	Executive function
Rey Complex Figure (copy)	Executive function
Digit Symbol	Processing speed
Controlled Oral Word Association	Processing speed
Hopkins Verbal Learning Test	Verbal memory
Brief Visual Memory Test	Visual memory

*Z*-scores were calculated from normative means and standard deviations for each neurocognitive test. Higher *Z*-scores represent better function. The composite cognitive *Z*-score was the average *Z*-score of all domains. Abnormality was −1.5 SD below the normative mean.^7^ A clinically significant change in cognition was pre-defined as a 0.5 SD change from baseline.^8^

### Statistical analysis

*t*-tests and Spearman’s correlation coefficients were used. Tests with multiple observations (e.g. individual cognitive domain score) had Bonferroni correction at *P* = 0.01.

## Results

Seventy participants signed informed consent of whom we assessed 42 at baseline and 34 after TAVR for TCD and 31 with neurocognitive assessment. Four failed the dementia assessment. There was a mean of 6.38 days between baseline assessment and TAVR. *Table 2* displays demographics. There were no demographic, baseline TCD, or neurocognitive differences between those who were and were not seen in follow-up.

**Table 2:** Patient demographics

Demographics	
*n*	42
Age mean (range)	78 (52–93)
Education mean [years (SD)]	14 (3.6)
Male [*n* (%)]	24 (59)
Race [Caucasian *n* (%)]	39 (93)
STS [PROM (mean (SD)] surgical mortality score	3.91 (1.87)
Valve area [cm^2^; mean (SD)]	0.75 (0.21)
Mean aortic valve gradient [mmHg (SD)]	46.87 (10.67)
LVEF (<50%)	2
Mean arterial pressure [mean (SD)]	92.21 (19.1)
Hx of stroke (%)	2
Hx of TIA (%)	2
Hx of hypertension (%)	90
Hx of diabetes (%)	43
Hx of dyslipidaemia (%)	69
Hx of Afib (%)	31
NYHA Class III (%)	8

STS (PROM), The society of thoracic surgeons predicted risk of mortality; LVEF, left ventricular ejection fraction; TIA, transient ischemic attack; Afib, atrial fibrillation; NYHA, New York State Heart Association.

**Figure 1:**
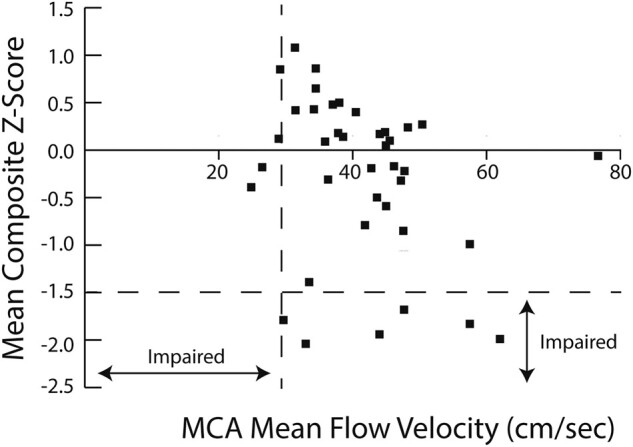
Mean composite *Z*-scores and cerebral blood flow before transcatheter aortic valve replacement. Abnormality: ≥1.5 SD below the normative means for cognition and for mean flow velocity. MCA, middle cerebral artery..

Before TAVR, average MFV was 42.36 cm/s (SD 10.17), with 2/42 having impaired CBF velocities. The mean composite cognitive baseline *Z*-score was −0.25 SD below normative mean. Neither the overall mean composite *Z*-score nor the individual domain scores were in the impaired range. Among individual patients, 6/42 were impaired. Two patients missed their pre-procedure cognitive assessments. *Figure 1* displays the composite *Z*-scores and MFV in the MCAs, along with the designation of impairment. There was a non-significant negative correlation (*r* = −03.11, *P* = 0.115). Of the six patients with impaired cognition, none had abnormal MFVs. Of the two patients with abnormal MFVs, neither had abnormal cognition.

Thirty-four patients returned for the post-TAVR follow-up; the mean follow-up period was 36 days. At follow-up, the average MFV was 41.59 cc/s (SD = 10.42), not statistically different from average baseline MFVs [*P* = 0.66 (−2.38 to 3.67)]. After TAVR, the mean composite cognitive *Z*-score was 0.17 SD above the normative mean, a statistically significant change from baseline but not meeting the 0.5 SD threshold for clinical improvement.

## Discussion

We found little impairment in MFV on TCD in the middle cerebral artery prior to or after TAVR. There was no correlation between baseline MFV and neurocognition. Although there was a statistically significant improvement in neurocognition from pre- to post-TAVR, the pre-established criterion for a clinically relevant change was not met. Thus, we found no evidence that AS has an impact on CBF and a corresponding cognitive effect.

That TAVR might increase cerebral flood flow was based on the premise that post-procedure cognitive improvement may have been from increased haemodynamics across the new aortic valve. Another study measured CBF of the total grey matter quantified by arterial spin labelling (ASL) with 3 T MRI.^9^ Their increase in perfusion was from 42.5 to 43.8 mL/100 g/min over the entire brain, findings similar to ours, despite improved cardiac output, which was not statistically significant (*P* = 0.41).

Cerebral blood flow is governed by autoregulation that expands and contracts vessels with alterations of perfusion and the extraction of a percentage of oxygen from the blood supply, which increases only when autoregulatory capacity has been exhausted.^10^ Our data showed normal-range MFV on TCD at baseline, suggesting that impaired flow across the stenotic aortic valve is not sufficient to exceed the brain’s autoregulatory capacity.

### Limitations

The *N* was relatively small, but our sample was representative of the TAVR population with intermediate risk. Our results may not generalize to more severe AS cases. There was no control group, but we could not ethically delay or preclude TAVR for whom there was a clinical indication for treatment. Little change on TCD after TAVR reduced the likelihood of measurement variability of blood flow. A third limitation was that the 30-day follow-up may not have been long enough to demonstrate a cerebral haemodynamic impact. We did not measure more subtle changes in cerebral haemodynamics, such as vasomotor reactivity, flow acceleration, or dynamic cerebral autoregulation, and we recognize that MVF is only a surrogate for cerebral perfusion.

Our goal was not to show the prevalence of cerebral haemodynamics of those undergoing TAVR but to determine whether CBF was independently related to AS and improved after valve replacement. These findings suggest that cerebral haemodynamic mechanisms are sufficient to maintain cognition in the setting of this disease.

## Data Availability

The data are available for sharing through a request to the corresponding author.

## Lead author biography

Ronald M. Lazar, PhD, FAAN, FAHA, holds the Evelyn F. McKnight Endowed Chair and is a Professor of Neurology and Neurobiology and Director of the UAB McKnight Brain Institute at the University of Alabama at Birmingham in the USA. His research, largely funded by the US National Institutes of Health, focuses on the relationship between neurocognition, and cardiovascular and cerebrovascular disease. Prior to joining UAB in 2017, he was a Professor of Neuropsychology in Neurology and Neurosurgery at the Columbia University College of Physicians & Surgeons in New York City and the New York Neurological Institute.
